# From Vibrations to Visions: Raman Spectroscopy’s Impact on Skin Cancer Diagnostics

**DOI:** 10.3390/jcm12237428

**Published:** 2023-11-30

**Authors:** Charlotte Delrue, Reinhart Speeckaert, Matthijs Oyaert, Sander De Bruyne, Marijn M. Speeckaert

**Affiliations:** 1 Department of Nephrology, Ghent University Hospital, 9000 Ghent, Belgium; charlotte.delrue@ugent.be; 2 Department of Dermatology, Ghent University Hospital, 9000 Ghent, Belgium; reinhart.speeckaert@uzgent.be; 3 Department of Laboratory Medicine, Ghent University Hospital, 9000 Ghent, Belgium; matthijs.oyaert@uzgent.be (M.O.); sander.debruyne@uzgent.be (S.D.B.); 4 Research Foundation-Flanders (FWO), 1000 Brussels, Belgium

**Keywords:** Raman spectroscopy, skin cancer, tumor differentiation

## Abstract

Raman spectroscopy, a non-invasive diagnostic technique capturing molecular vibrations, offers significant advancements in skin cancer diagnostics. This review delineates the ascent of Raman spectroscopy from classical methodologies to the forefront of modern technology, emphasizing its precision in differentiating between malignant and benign skin tissues. Our study offers a detailed examination of distinct Raman spectroscopic signatures found in skin cancer, concentrating specifically on squamous cell carcinoma, basal cell carcinoma, and melanoma, across both in vitro and in vivo research. The discussion extends to future possibilities, spotlighting enhancements in portable Raman instruments, the adoption of machine learning for spectral data refinement, and the merging of Raman imaging with other diagnostic techniques. The review culminates by contemplating the broader implications of these advancements, suggesting a trajectory that may significantly optimize the accuracy and efficiency of skin cancer diagnostics.

## 1. Introduction

The rising prevalence of malignant skin tumors, such as basal cell carcinoma (BCC), squamous cell carcinoma (SCC), and malignant melanoma (MM), is a critical and socially significant challenge faced by healthcare systems worldwide. The first two, along with various other less-common cutaneous neoplasms, fall under the classification of non-melanoma skin cancers (NMSCs). The urgency of this issue is further magnified when we consider the profound impact of early detection on patient outcomes. Detecting malignant skin tumors at an incipient stage significantly amplifies the likelihood of a favorable treatment outcome, diminishes the financial burden of treatment, and expedites the recovery process for the patient. The existing “gold standard” for diagnosis involves clinical inspection followed by biopsy and histopathology, which are invasive, costly, and require a significant amount of time.

Currently, a range of non-invasive diagnostic tools are at the disposal of healthcare practitioners. Techniques such as dermatoscopy, ultrasound skin scanning, cross-polarization optical coherence tomography (CP-OCT), and thermography are routinely employed in clinical settings to aid in the diagnosis of skin malignancies. However, these existing methodologies have certain limitations, particularly in terms of the accuracy of early diagnosis. The quest for more precise and early diagnostic techniques is an ongoing endeavor that seeks to bridge the existing gaps in skin cancer diagnostics.

In light of the above, Raman spectroscopy has unique potential. This innovative technique illuminates a sample and scrutinizes scattered light for energy shifts, thereby generating a molecular fingerprint. This fingerprint is instrumental in the precise identification and differentiation of various types of skin cancers, including MM and NMSCs. In studies, it has shown promise in its ability to discriminate between various skin cancers and precancerous conditions and non-malignant skin disorders. This discernment is pivotal, as it paves the way for more accurate diagnoses, which, in turn, play a crucial role in the devising of effective treatment plans. The application of Raman spectroscopy is not confined to diagnostic precision. The technique presents a non-invasive, real-time analysis that is indispensable for prompt decision making in a clinical setting. Moreover, its potential for integration with other diagnostic technologies presents a pathway toward a multimodal approach to skin cancer diagnostics. 

In this review, our goal is to clarify the fundamental concepts of RS, critically assess its current position in the field of skin cancer diagnostics, and prognosticate the potential advancements it could aid in the future. The discussion will traverse the evolution of Raman spectroscopy, from its fundamental principles to its practical applications in skin cancer diagnostics, and finally, to the promising horizon of opportunities it unveils for augmenting early diagnosis, thereby ameliorating patient prognosis. Through a comprehensive exploration, this review sheds light on how Raman spectroscopy could potentially reshape the landscape of skin cancer diagnostics, marking a significant stride toward early, accurate diagnosis and improved patient outcomes.

## 2. The Science behind Raman Spectroscopy

**Figure 1:**
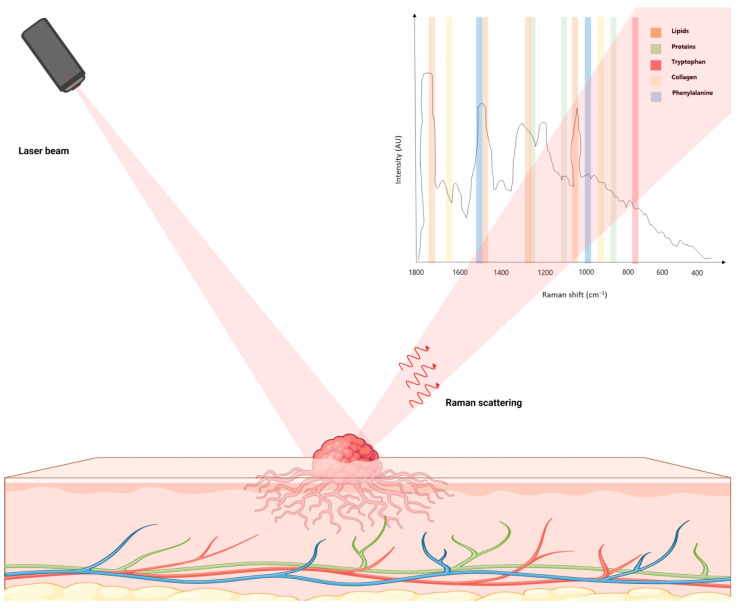
Application of Raman spectroscopy for identifying molecular vibrations in skin tissues, showcasing the unique Raman spectrum that facilitates the diagnosis and study of skin cancer.

Raman spectroscopy is a non-destructive analytical technique utilized to discern the molecular vibrations, crystal structures, chemical compositions, and physical properties of materials. Central to this technique is the phenomenon of the inelastic scattering of photons by molecules, which results in a shift in energy and, consequently, in the frequency of the scattered light. The change in energy, termed the Raman shift, is unique to the molecular vibrations within the sample, offering a ‘fingerprint’ that allows for the identification of molecules [1]. The procedure begins with a monochromatic light, typically a laser, being directed at the sample. When the light engages with the sample, the majority of the photons scatter elastically, maintaining their initial energy. However, a minor portion of the light scatters inelastically, resulting in photons either gaining or losing energy. This inelastic scattering leads to two distinct outcomes: Stokes scattering, where the scattered photons lose energy, and anti-Stokes scattering, where they gain energy. The energy variation between the incoming and scattered light corresponds with the vibrational transition energy within the molecule (Figure 1).

Nanotechnology, particularly surface-enhanced Raman scattering (SERS), has been highlighted for its potential in cancer nanomedical therapy because of its remarkable spatial precision, molecular selectivity, and stability, all key to reliable cancer detection [2,3]. The application of nanoparticles, particularly gold nanoparticles, to enhance Raman spectroscopic signals has also been acknowledged, and is pivotal for in vivo biosensing and skin cancer imaging [4,5]. The strength of the SERS signal is affected by the dimensions of the nanoparticles utilized. Typically, bigger nanoparticles can amplify the strength of the SERS signal [6].

Raman spectroscopy is simple to execute and necessitates no particular staining or preparation, facilitating real-time diagnosis and preventing disruptions to the surgical workflow [7]. Second, Raman spectroscopy delivers accurate diagnostic results within minutes, as opposed to traditional analytical techniques that take hours or even days [8]. Third, Raman spectroscopy is a non-invasive approach that is safe for patients. Additionally, its specificity at the molecular level renders it objective, facilitating the differentiation of early stage lesions [9]. Its high accuracy also reduces the need for additional costly tests to ensure correct diagnosis [10]. The use of Raman spectroscopy in medical diagnostics has been made more feasible with the development of specialized equipment to capture Raman scattering. This includes flexible fiberoptic Raman probes designed for clinical environments and portable spectrometers [11,12].

## 3. Raman Device Instrumentation

The instrumentation for Raman spectroscopy ranges from sophisticated laboratory setups to portable and handheld devices, adapted to various applications. In laboratory settings, a monochromatic laser serves as the light source, and the laser wavelength is a critical factor that affects the Raman signal and potential sample damage. The sample illumination and collection optics focus the laser light onto the sample and collect the scattered light, thereby influencing the sensitivity and resolution of the instrument. The spectrometer disperses this scattered light, and a detector, typically a CCD or photodiode array, captures and converts it into an electrical signal, which is controlled and analyzed using specialized software. In contrast, portable and handheld Raman devices are gaining popularity owing to their convenience, on-site analysis capability, and miniaturized components. These include smaller laser sources, compact optics, integrated spectrometers, and detectors, which often have the downside of reduced sensitivity and resolution. They boast user-friendly interfaces, are often battery-operated, and are equipped with wireless connectivity for field applications. Despite their versatility, these portable devices face challenges such as low sensitivity and resolution, specialized sample-handling requirements, and susceptibility to environmental factors. Regular calibration and maintenance are crucial, particularly for field devices. Recent advancements have seen the integration of Raman spectroscopy with smartphones and wearable technology, alongside the incorporation of artificial intelligence (AI) and machine learning to enhance the analysis of complex spectra, especially in less-controlled environments. The choice between laboratory-based and portable devices depends on the specific needs of the application, considering factors such as the need for portability, the sample nature, and the required analysis detail [13,14,15,16,17].

## 4. Raman Spectroscopy in Skin Cancer Diagnostics

### 4.1. Differentiation of Skin Cancer from Normal Tissue and between Skin Tumor Types

#### 4.1.1. Cell Lines

Raman spectroscopy allows for the detection of tumor cells and their adjacent stroma within the tissue context. It serves as an effective non-invasive instrument for differentiating between melanocytes and MM cells and can be utilized to ascertain the mutation status of cells (*BRAF* and *NRAS* mutations), examine the particular type of cell death in MM cells, and forecast the responsiveness of MM cells to anticancer drugs [18]. Through tracking the intensity levels of two separate Raman peaks at 752 cm^−1^ and 785 cm^−1^, the distribution characteristic of tryptophan-rich proteins and nucleic acids are observed, each at a subcellular level of detail. These specific Raman indicators are useful in distinguishing between standard human epidermal keratinocytes and dermal fibroblasts, and in separating all cancerous cells from both of these normal cell types. The initial application of these distinct intracellular nucleic acid and tryptophan Raman markers for diagnosing skin cancer was showcased in a three-dimensional skin model of SCC [19]. 

Confocal Raman spectroscopy holds the capability to distinguish between various cell lines. Notable disparities were observed between the spectra of HaCaT cells and SCC cells, melanocytes, and MM cells, as well as between all normal cells (comprising HaCaT cells and melanocytes) and all tumor cells (encompassing SCC and MM). The separations exhibited an approximate sensitivity and specificity of 90% across the board [20]. 

A method that integrates fiberoptic tweezers with RS for the swift identification of MM demonstrated accurate categorization of 150 Raman spectra from 30 cells across mutant, wild-type, and drug-resistant MM cell lines. The accuracy rates achieved were 92%, 94%, and 97.5%, respectively, following the implementation of a principal component analysis (PCA) and a support vector machine (SVM) classification model [21].

#### 4.1.2. Clinical Studies

A meta-analysis evaluated the precision of Raman spectroscopy in distinguishing skin cancer from healthy tissue, encompassing 12 studies [7,22,23,24,25,26,27,28,29,30,31,32] with 2461 spectra included (Table 1) [33]. RS showed more effective results in ex vivo detection than in vivo detection. In detecting BCC ex vivo, the technique achieved a sensitivity of 0.99 (95% CI: 0.97–0.99) and a specificity of 0.96 (95% CI: 0.95–0.97), with an area under the curve (AUC) of 0.98. In contrast, in vivo BCC detection showed a sensitivity of 0.69 (95% CI: 0.61–0.76) and a specificity of 0.85 (95% CI: 0.82–0.87), with an AUC of 0.92. In detecting MM ex vivo, the sensitivity was 1.00 (95% CI: 0.91–1.00), and the specificity was 0.98 (95% CI: 0.95–1.00), with an AUC of 0.99. In vivo MM detection achieved a more balanced sensitivity (0.93) and specificity (0.96). In the detection of SCC ex vivo, the sensitivity was 0.96 (95% CI: 0.81–1.00), and the specificity was 1.00 (95% CI: 0.92–1.00). However, in vivo SCC detection recorded a sensitivity of 0.81 (95% CI: 0.70–0.90) and a specificity of 0.89 (95% CI: 0.86–0.91). In vivo detection might provide less detailed information about the lesions due to the restricted time available for acquiring Raman images [7]. On the other hand, ex vivo Raman imaging, conducted via scanning across lesion surfaces, can reveal the spatial distribution of various tissue structures. Nonetheless, this method is not practical for clinical use due to its time-consuming nature, and it is difficult for patients to remain completely motionless, a requirement for obtaining images with a high spatial resolution [7,22]. The precision of Raman spectroscopy in differentiating MM from healthy tissues is higher than its ability to distinguish between BCC and SCC. This greater accuracy can be attributed to the different cellular origins of these cancers; whereas BCC and SCC both stem from malignant keratinocytes, MM is derived from melanocytes [34,35,36]. Due to compositional differences, the spectral disparities seen in the spectra of MM are markedly more distinct and appear in varying wavenumber ranges compared to those of BCC and SCC. In contrast, the spectra of BCC and SCC display prominent variations but within comparable wavenumber ranges [37]. Eight primary spectral signatures related to the cell nucleus, keratin, elastin, ceramide, melanin, collagen, triolein, and water are identified as vital for characterizing the spectral features in the Raman spectroscopy analysis of human skin [38]. In addition, spectral disparities were primarily seen as a heightened presence of symmetric CH_2_ stretching vibrations associated with lipids in MM relative to benign melanocytic lesions [39]. A study involving 145 spectra from biopsy samples of normal, BCC, and SCC cutaneous tissues, conducted with a near-infrared Raman spectrometer (NIR-RS) connected to a Raman probe, pinpointed key biochemicals such as actin, collagen, elastin, and triolein as indicative of the spectral properties of skin tissues. The classification model successfully distinguished normal tissues from BCC and MM tissues by evaluating the relative presence of collagen III, elastin, and melanin, using Euclidean distance as a distinguishing factor [32]. Another research project showcased the capability of PCA and Euclidean distance to differentiate between the Raman spectra of BCC and MM skin fragments and healthy tissues in vitro. The principal component vectors provided critical spectral data essential for grasping the biochemical differences in tissues linked with pathological states [22]. 

Raman spectroscopy has been employed to identify spectral variances between normal skin tissues and those affected by BCC [28,40,41,42,43,44,45,46]. Significant spectral differences between these samples were noted in the wavenumber ranges of 800–1000 cm^−1^ and 1200–1300 cm^−1^, corresponding to vibrational bands from lipids and proteins (C-C bonds and amide III, respectively). For classifying tissue types, a diagnostic approach based on PCA and Mahalanobis distance was applied to the scores from the principal component vectors PC1 and PC2. This method demonstrated a sensitivity and specificity of 89% and 93%, respectively, in the training group, and 96% and 92% in the prospective group. Additionally, a basic biochemical model assessing collagen levels achieved a sensitivity and specificity of 95% and 83% in the training group, and 87% and 92% in the prospective group [40]. Dual-vibration resonance frequency within coherent anti-Stokes Raman scattering assessments has been employed to produce pseudo-hematoxylin-and-eosin (H&E)-stained ex vivo microscopic images of BCC human skin specimens, facilitating their detection and identification [47].

Furthermore, biological insights related to SCC at the molecular level can be clarified via Raman microspectroscopy. In one study that used H&E-stained pathological tissue sections, there was a notable decline in the collagen-related Raman peak intensities (853, 936, and 1248 cm^−1^), but there was a notable increase in the DNA (720, 1327 cm^−1^) and lipid (1305 cm^−1^) peak intensities inside the cancerous lesions. These findings are consistent with the theory that SCC starts in the epidermis and gradually spreads to the dermis [48]. The pathological characteristics of SCC tissues were discerned using stimulated Raman scattering microscopy. The observed morphological attributes of SCC within stimulated Raman scattering microscopy aligned well with the diagnostic criteria ascertained through histological analysis. Moreover, stimulated Raman scattering microscopy has demonstrated superior cellular contrast relative to confocal microscopy [55].

Prior to a histological inspection of tissue, NMSCs can be effectively diagnosed using the non-invasive method known as spatially offset Raman spectroscopy (SORS). With a bias toward the various levels of the epidermis, where NMSCs first develop and proliferate, this technique is used to gather Raman photons and examine deeper skin tissue layers. In a study, 22 skin samples were subjected to conventional back-scattering and a defocused modality (with and without a spatial offset) to acquire ex vivo Raman data. The spectrum data and the related histopathology data were analyzed in order to find potential predictors for lesion detection. PCA revealed that while wavenumbers 507, 631, 772, 870, 1076, 1189, 1268, 1300, 1435, and 1531 cm^−1^ were linked to SCC, wavenumbers 937, 1003, 1125, 1339, and 1640 cm^−1^ were not [49]. Certain spectral characteristics distinguishing the two subtypes are bands associated with phenylalanine (at 1003 and 1339 cm^−1^), skeletal (at 937 cm^−1^), and amide I (at 1640 cm^−1^) collagen vibrations, which are notably more pronounced in the SCC type [27,56,57]. Because of the prevalence of hyperkeratosis, SCC has been shown to have a positive connection with the phenyl ring content [58]. On the other hand, spectral bands moving in the opposite direction are attributed to tryptophan (at 772 cm^−1^), lipids (at 1076 cm^−1^, 1300 cm^−1^, and 1650 cm^−1^), and proteins (amide III at 1268 cm^−1^ and hydroxyproline at 870 cm^−1^), and are prominent in the BCC group [27,56,57]. The spectral bands at 1339 cm^−1^ and 1076 cm^−1^ have been identified as being related to nucleic acids, bases (adenine), and backbones, as well as lipids and proteins [56,59]. The BCC spectra show a stronger presence of the 1743 cm^−1^ band. This may be explained by the lipid contribution from the ester groups in the glycerol heads of triacylglycerol in the adipose tissue below the dermis in the C-O stretching mode [49,60], or it could also suggest a higher amount of melanin owing to more pigment in the BCC patient group, as a subgroup of pigmented BCCs exists [57]. The results indicated a favorable relationship between lipid content and BCC and between protein content and SCC. Lipid spectrum features were noted in the BCC and SCC groups, as well. More precisely, PC2 loadings displayed negative peaks (characteristic of the BCC group) at 1659 cm-³ and 1080 cm^−3^, which are linked to unsaturated lipids, primarily triolein from adipocytes, and fall between 1260 and 1310 cm^−1^ [49]. The esterification of glycerol’s three hydroxy groups with oleic acid forms triolein, a triglyceride important for epidermal skin [61]. Conversely, positive peaks (characteristic of the SCC group) at 1128 and 1283 cm⁻^1^ are linked to the phospholipids sphingomyelin and phosphatidylcholine from the cell membrane, as well as saturated fatty acids of ceramides from the epidermis. Statistically significant differences between the two pathological groups were revealed via estimating the ratio of signature bands from unsaturated (1080 cm^−1^) and saturated (1128 cm^−1^) lipids across all measurement modes [49]. These findings align with those of previous studies [58], where the absence of ceramide in the skin is believed to trigger the mutation of basal cells in the epidermis, initiating BCC growth [62]. Regarding collagen content, PCA highlighted a heightened presence of bands at 937 cm^−1^ and 1640 cm^−1^, which are attributed to the proline and hydroxyproline backbone and the amide I vibrations of collagen, respectively, in the SCC spectra compared to the BCC spectra [49]. While the abundance of collagen in SCC is yet to be extensively studied, a few studies have confirmed a negative correlation between collagen and BCC, supporting the notion of a lower abundance of connective tissue, and thereby collagen, in BCC-affected skin tissue [63,64]. An inverse biophysical model for skin cancer delineated the proficiency of RS in elucidating the biophysical underpinnings required for the precise diagnosis of diverse cutaneous cancer types. Collagen and triolein have emerged as paramount biomarkers, epitomizing the spectral discrepancies between MM and dysplastic nevi, and between NMSCs and healthy skin tissue [65]. Multiple-factor analysis showed a significant grouping of factors based on age and sex across all modalities after additional correlation with patient data. Specifically in the defocused modality (zero and 2 mm offset), pathology-based extra clustering took place [49]. 

The utilization of normal-subtracted preprocessing on Raman spectra considerably augments the diagnostic advantages of distinguishing between normal skin, benign dermatitis, BCC, SCC, and actinic keratosis. Spectral peaks corresponding to proteins and lipids manifested elevated levels in neoplastic tissues relative to non-neoplastic tissues, with lipid concentrations being pronounced in BCC and keratosis sites compared to non-neoplastic sites. BCC exhibited a higher prevalence of nucleic acid features than SCC, whereas SCC displayed elevated levels of proteins (specifically elastin and collagen) relative to the other tissue types examined. Features indicative of inflammation (such as blood cells) were discernible in benign lesions and actinic keratosis. The discrimination model predicted using partial least-squares discriminant analysis (PLS-DA) elucidated that a 90.3% accuracy rate in tissue differentiation could be achieved when deploying mean-subtracted spectra, a significant enhancement from the 75.1% accuracy observed with non-subtracted spectra. Furthermore, the discrimination of non-neoplastic tissues from pre-neoplastic and neoplastic tissues showed an accuracy escalation of 92.5% with normal-subtracted spectra as opposed to 85.3% with non-subtracted spectra [50]. Utilizing wavenumber-selection-centric analysis can significantly enhance the diagnostic specificity of Raman spectroscopy for the detection of skin cancer. In a merged cohort encompassing 645 verified lesions from 573 patients, including skin cancers, precancers, and benign skin lesions, the deployment of the least-absolute-shrinkage-and-selection-operator (LASSO)-based wavenumber selection coupled with PCA and general discriminant analysis (GDA) increased the area under the receiver operating characteristic curve (ROC) metrics from a range of 0.86–0.89 to 0.89–0.91. Simultaneously, it increased the diagnostic specificity from a span of 0.17–0.65 to 0.20–0.75, given fixed sensitivities ranging between 0.99 and 0.90. Comparable enhancements in diagnostic performance were observed when stepwise regression and PLS-based analyses were employed [51]. The multivariate curve resolution alternating least-squares (MCR-ALS) technique was applied to analyze 1000 in vivo Raman spectra. These spectra consisted of 540 normal skin samples, 113 actinic keratosis, 122 BCC, 67 MM, and 158 pigmented nevi. For the comparison between BCC and normal skin, as well as the classification of all diseases including neoplasms, the highest ROC AUC achieved was 0.772 (95% CI: 0.720–0.823). The most effective differentiation between BCC and MM was observed with ROC AUCs of 0.700 (95% CI: 0.626–0.773) and 0.690 (95% CI: 0.623–0.757), respectively. When distinguishing between malignant neoplasms and benign neoplasms, the highest ROC AUC obtained was 0.653 (95% CI: 0.602–0.705). The ROC AUC for the discrimination models between MM and pigmented nevus was 0.656 (95% CI: 0.574–0.738) [52]. In another study, 44 biopsy specimens encompassing MM, dysplastic nevi, and compound nevus tumors were examined using Raman spectroscopy imaging in conjunction with the MCR-ALS algorithm. This methodology facilitated the non-destructive localization and quantification of total eumelanin along with the 5,6-dihydroxyindole-2-carboxylic acid subunit content within cutaneous lesions. A heightened ratio of 5,6-ihydroxyindole to 5,6-dihydroxyindole-2-carboxylic acid was discerned in dysplastic nevi compared to MM and compound nevus lesions. This elevated ratio observed within dysplastic nevi might elucidate its proclivity to progress into MM, potentially owing to the altered modulation of oxidative stress mechanisms. The investigative approach yielded a sensitivity and specificity of 100% and 94.1%, respectively, for the identification of dysplastic nevi lesions. Moreover, diagnostic sensitivities and specificities of 82.4% and 92.9% were attained for dysplastic nevi and MM samples, respectively [53].

The use of silver-coated silicon nanowires (Ag/SiNWs) as a RS platform made it easier to distinguish between genomic DNA (healthy and malignant, respectively) from normal human skin cells and MM cells. A dense array of Ag/SiNWs was immediately coated with DNA samples in aqueous droplets, which were then allowed to evaporate before being subjected to Raman mapping. Using PCA on the collected Raman spectra in the droplets allowed the differentiation of malignant and healthy cell DNA, with fewer than 2% false positives and no false negatives. This differentiation process occurred using Raman bands that are closely linked to the interaction between DNA and nanowires, rather than particular DNA sequencing examination. The underlying reason for this observed difference may be related to the structural differences and/or different charge properties that are present in the DNA of healthy and malignant cells. Following the evaporation of water, these effects cause a divergent arrangement of molecules to be adsorbed onto the nanowires [54].

Advanced Raman techniques such as stimulated Raman spectroscopy (SRS) and coherent anti-Stokes Raman spectroscopy (CARS) are recognized for their non-linear imaging capabilities, enabling rapid data acquisition and near-video-rate imaging speeds. These attributes provide a robust platform for enhanced optical analysis and screening of skin tissues [66]. SRS and CARS have been instrumental in identifying subtle differences in lipid composition and molecular content, offering valuable tools for diagnosing and characterizing skin cancers. The ex vivo characterization of NMSCs was carried out using a comprehensive optical multimodal methodology that includes the concurrent application of CARS, second-harmonic generation, and two-photon excited fluorescence. Both BCC and SCC exhibited a lack of second-harmonic generation, with an increased two-photon excited fluorescence signal within cancerous tissue. Notably, a discernible difference in the coherent anti-Stokes Raman scattering imagery between BCC and SCC was documented. The regions affected by BCC displayed a subdued coherent anti-Stokes Raman scattering signal, in contrast to the pronounced coherent anti-Stokes Raman scattering signal observed within SCC nests relative to the adjacent tissue. Beyond mere tumor nest delineation, this multimodal imagery evaluation provides prognostically relevant insights into lipid metabolism, bioenergetics, and tumor–stroma interactions. Moreover, the distinctive morphological attributes of the cancer types, as identified using H&E staining, were discernible. These include a palisade-shaped tumor border, retraction artifacts, and core necrosis in BCC, and increased cell density, larger nuclei, and a changed nuclear–cytoplasmic ratio in SCC. The combined data greatly assist in differentiating between healthy skin and NMSCs, providing a basis for discriminating between BCC and SCC and a way to evaluate specific characteristics of tumor biochemistry [67].

### 4.2. Combination with Imaging Techniques

A myriad of cutting-edge Raman techniques encompassing coherent, stimulated, and resonance Raman techniques, when interfaced with traditional or confocal applications of Raman spectroscopy, lay the groundwork for the amplified optical analysis and scrutiny of skin tissues, instrumental in disclosing the intricate molecular landscape of skin cancers and fostering a more precise and early diagnosis. The expansive applications and ongoing advancements in this integrated methodology underscore its potential in transforming skin cancer diagnostic procedures, thus contributing to the overarching goal of improving skin cancer management and patient care.

Raman spectroscopy, when amalgamated with imaging techniques, augments the diagnosis of skin cancer through the procurement of high-resolution images alongside the molecular data of the scrutinized skin tissues [68]. Raman spectroscopy paired with imaging allows for the creation of detailed maps that reveal both tissue morphology and the distribution of molecules. This integrated approach not only aids in early skin cancer detection by identifying subtle molecular changes but also informs treatment planning by assessing lesion extent and monitoring treatment responses. Additionally, it has the potential to reduce the need for invasive biopsies, improve patient comfort, and minimize scarring [68]. A significant advancement in this field is the emergence of real-time Raman spectroscopy systems harmonized with imaging for in vivo skin cancer diagnosis. In a specific investigation, a real-time Raman spectroscopy system was deployed to appraise benign and malignant skin lesions across a multitude of patients, markedly aiding in the prompt and accurate diagnosis of skin cancer and furnishing vital insights into the molecular deviations linked to malignancies [69]. Moreover, the inception of SERS has surfaced with high sensitivity and spectral resolution, although it encounters hurdles such as inadequate molecular generality and relatively subpar reproducibility [70]. The incessant evolution of Raman spectroscopy methodologies in tandem with imaging modalities is catalyzing the transition from bench to bedside, inching the technology closer to clinical applicability [71]. 

A valuable player from this insight is the increasing recognition of hyperspectral imaging (HSI). HSI is a robust technology capable of capturing continuous spectral data, offering more detailed color information and discerning subtle characteristics of light sources and scenes. It combines 2D spatial pixels with spectral data to create a 3D data cube. This fusion of spatial and spectral information enables the quantification of the physical and chemical properties of materials. Its primary significance lies in biomedical contexts, encompassing tasks such as analyzing cancerous tissues, identifying substances, monitoring physiological parameters, and facilitating real-time disease diagnosis. However, the dynamic nature of biological tissues necessitates high-resolution hyperspectral systems for real-time imaging to track tissue changes continuously [72]. Hyperspectral systems offer a multitude of advantages over traditional spectral systems. These systems, which are well known for their ability to capture a comprehensive range of spectral data, provide a quantum leap in data acquisition and analysis. They excel in offering a high spectral resolution, allowing for the discernment of subtle differences that often escape conventional spectral systems. In addition, hyperspectral imaging integrates spatial information, resulting in 3D data cubes that enhance context awareness and object recognition. Material identification and characterization benefit significantly from their capabilities, spanning applications from mineral identification to land cover classification. Moreover, hyperspectral systems contribute to non-invasive data acquisition in medical contexts, enabling real-time imaging and dynamic monitoring. Their versatility extends across diverse fields, including remote sensing, precision agriculture, and security. Furthermore, they serve as invaluable research tools, aiding the exploration of molecular and biochemical complexities across various scientific domains. In summary, hyperspectral systems usher in a new era of data richness, enabling enhanced insights and capabilities across a wide spectrum of applications [73,74,75,76,77].

### 4.3. Multimodal Approaches

Merging Raman spectroscopy with other spectroscopic methods such as fluorescence or infrared (IR) spectroscopy augments data richness, enabling a more thorough investigation of skin tissues. For instance, IR spectroscopy can reveal alterations in protein secondary structures within MM tissues, thus complementing the molecular insights gained from Raman spectroscopy [78]. While Raman spectroscopy primarily delves into the molecular vibrations and chemical composition of proteins, IR spectroscopy focuses on the absorption of IR light by chemical bonds, including those within proteins. This tandem approach offers a comprehensive perspective on alterations in protein secondary structures, which are frequently observed in MM and other diseases. In particular, the sensitivity of IR spectroscopy to amide bonds in proteins, integral to secondary-structure elements like α-helices and β-sheets, is invaluable. By combining the two techniques, researchers gain insights into both the specific amino acid residues and molecular vibrations associated with protein conformation provided via Raman spectroscopy, as well as the broader changes in overall protein secondary structures detected via IR spectroscopy [78].

#### 4.3.1. Multi-Wavelength Raman/Photoluminescence Microspectroscopy

Using wavelengths of 532, 785, and 1064 nm in multiwavelength Raman and photoluminescence (PL) microspectroscopy, in vitro samples of healthy skin, BCC, and SCC were distinguished from one another. Following a standardized process for baseline correction and smoothing, the resulting Raman spectra showed significant bands predominantly corresponding to different vibrational modes of membrane lipids chosen as biomarkers for the first time, with a high degree of similarity. These bands’ intensity ratios were useful in differentiating between benign and malignant skin cancers. Two novel wavelength-independent spectral criteria were identified through the use of MCR-ALS analysis of the Raman and PL spectra of skin neoplasms at all wavelengths to confirm the proper selection of spectral bands. The importance of triolein, in particular, as a biomarker for cell membrane lipids was brought to light through this investigation. With classification rates of 96.2%, 77.8%, and 90%, respectively, it was possible to reliably identify normal skin, BCC, and SCC using an excitation wavelength of 532 nm. By comparing the results to normal skin, dermatology specialists using other techniques like thermometry, ultrasound skin scanning, cross-polarization optical coherence tomography, spectroscopy, and terahertz range visualization were able to achieve similar sensitivity (96.2%) and specificity (94.7%) in detecting all tumors. With a 100% classification rate using a 785 nm excitation wavelength, SCC could be distinguished from both normal skin and BCC. However, due to their close resemblance, it was difficult to separate BCC from normal skin (with a true positive rate of 47.1%). The sensitivity and specificity of this wavelength were 84% and 72.7%, respectively. The wavelength that was designated allowed for accurate in vitro distinction between normal skin and malignancies, and the excitations at 532 nm and 785 nm helped to reliably identify BCC and SCC. Improving the suggested technique for the integrated analysis of multi-wavelength Raman data could improve the detection rate of malignant skin tumors and solve the drawbacks of using each excitation wavelength separately. Ultimately, by using an excitation wavelength of 1064 nm, all data could be divided into two groups: normal skin and cancers, with corresponding sensitivity and specificity of 89.5% and 96.9%. This was explained by the fact that there were no discernible variations in the lipid main bands in the BCC and SCC Raman spectra [79].

#### 4.3.2. Raman Spectroscopy and Machine Learning

The synergy between Raman spectroscopy and machine learning algorithms has been explored for skin cancer detection, with studies demonstrating the potential of spectral classification for identifying BCC from tissue samples. Furthermore, data augmentation techniques have been shown to enhance skin cancer tissue classification accuracy, indicating the robustness of integrating machine learning with Raman spectroscopy [80]. The coupling of Raman spectroscopy with artificial intelligence algorithms also enables real-time spectral data analysis with high specificity and sensitivity, demonstrating significant advancements in this domain [81]. In one of the first studies, the classification of skin lesions, based on in vitro Raman spectroscopy and using a neural network classifier, exhibited a classification accuracy of 80.5% ± 5.3% for MM when tested on a dataset encompassing 222 cases and 5 different lesion types. This rate is comparable to the diagnostic accuracy of trained dermatologists using visual inspection. Additionally, the classifier demonstrated an outstanding classification rate of 95.8% ± 2.7% for BCC. The overall accuracy rate for skin lesion classification stood at 94.8% ± 3.0%. The analysis revealed that small distinctive bands within the spectrum, which correspond to specific lipids and proteins, harbor discriminative information leveraged via the network for skin lesion diagnosis [82].

Integrating Raman spectroscopy with reflectance confocal microscopy imaging enhanced via deep learning is a promising tool for the detection of BCC and guidance in tumor resection surgery. Reflectance confocal microscopy serves as an auxiliary optical imaging method, providing non-invasive insights into skin structures in vivo at the subcellular level of detail [83]. It has progressed into clinical practice for the non-invasive diagnosis of skin cancer [84]. Through the employment of transfer learning to train two convolutional neural network (CNN) models for the identification of hair structures and the epidermis in RCM images, 52% of the false positives generated via Raman spectroscopy were eliminated, while 100% sensitivity was retained. The reflectance confocal microscopy models were proficient in accurately discerning images that exhibited circular arrangements of hair structures and ribbon formations in the epidermis [85].

The accuracy of classifying skin tumors through CNN utilizing Raman spectrum analysis is superior to, or on par with, the diagnostic accuracy of trained dermatologists. The spectra from 617 skin neoplasms (across 615 patients, including 70 MM, 122 BCC, 12 SCC, and 413 benign tumors) obtained using an in vivo portable Raman setup were classified. In the implementation of CNN, area under the receiver operating characteristic curve (ROC AUC) values of 0.96 (95% CI: 0.94–0.97), 0.90 (95% CI: 0.85–0.94), and 0.92 (95% CI: 0.87–0.97) were obtained for the classification of (a) malignant versus benign tumors, (b) MM versus pigmented tumors, and (c) MM versus seborrheic keratosis, respectively. Hence, the CNN analysis showed a higher efficiency than the projection on latent structure discriminant analysis (PLS-DA). The accuracy of the automatic analysis using the proposed CNN-based method surpassed that of general practitioners and trainees, and was either comparable to, or exceeded, the accuracy of trained dermatologists. The increased accuracy observed in the CNN implementation could be attributed to a more accurate consideration of low-intensity Raman bands amidst the pronounced autofluorescence background [86].

By focusing on the well-known “Raman biological fingerprint region” (800–1800 cm^−1^), machine learning models demonstrated great performance (AUC: 0.98, 95% CI: 0.97–0.99) in the classification of Raman spectra of human skin tissues between MM and benign melanocytic nevus. The protein bands amide I (1640–1680 cm^−1^), amide III (1220–1300 cm^−1^), and n(C-C) stretching (probably in the amino acids proline and hydroxyproline, 928–940 cm^−1^) were the key differences between benign and malignant spectra. Other bands that were indicative of lipids included CH_2_-scissoring vibration (1420–1450 cm^−1^) and -(CH_2_)n- in-phase twist vibration around 1300 cm^−1^. Furthermore, possible polysaccharide structural changes were found in the 840–860 cm^−1^ range. They constructed a high-performance model with a minimized spectral range (896–1039 cm^−1^). The critical region for the model’s decision making was 915–924 cm^−1^, followed by 1031–1039 cm^−1^. This indicates that only one smaller portion of the biological fingerprint Raman region is needed to reliably distinguish benign from malignant skin lesions. These microscopic zones were identified for the C-C stretch of proline, hydroxyproline, glucose, and lactic acid, as well as the C-H plane bending mode of phenylalanine, after a biochemical comparative investigation with well-established Raman table data. The biochemical changes pertaining to these components are associated with the progression of MM and suggest alterations in the structural collagen of the stroma surrounding the lesions [87].

Different Raman shift intensities and matching component abnormalities could be signs of MM. Analyzing the tissue composition in experiments could confirm this conclusion, which might be useful for upcoming research on the pathophysiology of cancer [80]. Given that diseases like cancer are known to induce genetic mutations and damage to DNA, it is possible that the emphasis on the 497 cm^−1^ Raman shift and its correlation with DNA components stems from these genetic alterations [81]. Moreover, the proliferation of MM cells in vitro can be efficiently inhibited through the regulation of the glutamate and cystine levels associated with MM, as demonstrated by the Raman shifts seen in the 515–545 cm^−1^ range [82]. One of the known contributing factors to medication resistance in some MM cells is the interaction between proteins and cell membranes [83]. Proteins and membrane phospholipid heads have their corresponding peaks at 717–719, 1160, 1244, and 1271 cm^−1^. The Raman shifts at 662, 759, 919, 1160, and 1360 cm^−1^ are associated with particular signals related to the overall protein content and amino acids such as proline, tryptophan, and tyrosine. These changes have been identified as putative indicators of the advancement of MM [84,85,86,87]. The Raman Transformer (RaT) model’s visualization results highlight the distinct Raman shifts and the parts that go along with them in MM classification screening. This emphasizes how important it is to distinguish MM cells from normal cells using certain amino acids, proteins, membrane phospholipid heads, and DNA. Understanding the principles behind these factors can provide important insights into the underlying mechanisms of MM [88,89,90,91,92,93,94,95], opening the door for the creation of novel therapeutic approaches [88].

The RaT model integrates Raman spectroscopy with a Transformer encoder, targeting the differentiation of Raman spectra between MM and normal tissues [88]. When it comes to handling long-distance dependencies, Transformers has demonstrated clear advantages over CNN [96]. Since Raman spectra are one-dimensional long-range data, the transformer encoder has become a more appropriate option for Raman spectrum classification. In contrast to traditional techniques for categorizing Raman spectra [97,98,99], the self-attention mechanism, a notion taken from the field of natural language processing, is used by the RaT model to capture correlations between vectors. It does this by segmenting the Raman spectrum data into blocks and treating each block as a word vector. The model can concentrate on both the global and local aspects in the data thanks to this feature. It has been demonstrated that the self-attention mechanism improves performance in deep learning applications, especially when dealing with large numbers of one-dimensional data. This invention addresses a number of issues: it overcomes the shortcomings of CNN in processing one-dimensional data, rises to the challenge presented by recurrent neural networks, and removes the necessity for manual feature analysis, which is evident in conventional machine learning and frequently fails to properly exploit huge datasets. Furthermore, when tested on datasets larger than the CNN model, the RaT model demonstrated better learning and anti-noise capabilities. RaT performed comparatively better in categorizing the Raman spectra of MM and normal tissues compared to other classification methods, which included PCA + Neural Network, CNN, and RNN, using the same dataset as a benchmark. With an accuracy of 99.69%, sensitivity of 99.86%, and specificity of 99.48%, it attained impressive numbers [88]. 

Deep learning, in conjunction with SERS spectroscopy, provides a powerful method for MM classification that greatly advances the analysis of, and research into, MM. A total of 20,000 SERS spectra were collected utilizing a planar solid SERS substrate containing positively charged AuNPs from 10 cell lines, include melanocytes and MM cells [100]. MM cells exhibit heightened spectral intensities at 645 cm^−1^ (C-C twisting mode of tyrosine) [101], 947 cm^−1^ (proline C-C stretching mode), 1030 cm^−1^ (protein C-C stretching, phenylalanine C-H stretching) [102], 1453 cm^−1^ (C-H bending mode of structural proteins) [103], and 1582 cm^−1^ [pyrimidine ring (nucleic acids) and heme protein] [104]. To aid in the classification of melanocytes and MM cells, as well as wild-type and mutant MM cells with *BRAF* mutations and MM cells with different degrees of treatment resistance, a number of CNN classification models were created. With a maximum accuracy of 100%, the approach clearly outperformed the PCA-K-nearest neighbor (KNN) approach. Proteins were responsible for the majority of the spectrum intensity changes that were seen at 1030 cm^−1^, 1157 cm^−1^, 1582 cm^−1^, and 1632 cm^−1^. The efficacious activity of the drug was connected to variations in peak intensity. The first decrease in peak intensity may be due to drug-induced cell death, whereas the second increase in intensity may be the result of drug resistance developing in the remaining sensitive cells and the removal of dead cells. Significant differences in Raman peaks were mostly concentrated around 1030 cm^−1^, possibly as a result of the susceptibility of phenylalanine to cell death [100]. This is consistent with the pattern of alterations noted by Zhang et al., who dynamically evaluated the treatment resistance and heterogeneity of BGC823 gastric cancer cells using single-cell Raman spectra [105]. The integration of deep learning and Raman spectroscopy in cancer diagnosis can lead to instances where certain tumor samples are challenging to classify accurately, potentially resulting in a lower accuracy compared to diagnoses by experienced medical professionals. This is because of various factors, including the considerable variability in tumor molecular composition, the limited availability of diverse and representative data for training, the inherent complexity of cancer biology with its various subtypes, and the sensitivity of Raman spectroscopy to factors such as sample preparation and environmental conditions. Moreover, medical doctors bring their clinical expertise and contextual information into the diagnostic process, which deep learning models may not offer. Researchers are actively working to address these challenges, striving to enhance the robustness and generalizability of deep learning models in medical applications, while acknowledging the value of combining automated methods with expert medical judgment to achieve more accurate cancer diagnoses. Ongoing research and the expansion of well-curated datasets hold promise for improving the performance of these integrated approaches in Raman-spectroscopy-based cancer diagnostics [106].

#### 4.3.3. Raman Spectroscopy and Optical Coherence Tomography

Hybrid systems amalgamating Raman spectroscopy with diagnostic modalities, such as OCT or high-frequency ultrasound, provide both morphological and biochemical insights, which are instrumental for a comprehensive assessment of skin lesions [107]. A combined module integrating cellular-resolution OCT and near-infrared Raman spectroscopy has been developed to differentiate various skin cancer cells from normal cells [108,109,110,111]. This module achieves micron-level three-dimensional (3D) spatial resolution and the capability to spectroscopically determine chemical components. Spatial and spectroscopic characteristics were used to distinguish between five types of skin cells: keratinocytes (HaCaT), the SCC cell line (A431), the BCC line (BCC-1/KMC), primary melanocytes, and the MM cell line (A375) [112]. Several critical cellular properties occur when OCT and Raman spectroscopy are combined. The first and foremost is cell morphology, where OCT excels in providing high-resolution images that reveal distinctions in shape, cellular density, and tissue architecture between cancerous and normal cells. Furthermore, variations in the refractive index, as captured using OCT, can signify changes within cancer cells, often linked to shifts in the nuclear-to-cytoplasmic ratios. NIR-RS complements this by delving into the chemical composition of cells. It unveils variations in biomolecules, such as proteins, lipids, and nucleic acids, all of which differ in their presence and distribution in cancerous versus normal cells. Additionally, Raman spectroscopy identifies specific cellular biomarkers, highlights metabolic alterations, and pinpoints changes in biomolecules, such as DNA, RNA, and proteins, collectively contributing to a comprehensive assessment of cellular characteristics. This integrative approach harnesses the power of both modalities to enhance diagnostic precision in distinguishing skin cancer cells from their healthy counterparts [38,113,114]. In one study, aspects like the cell volume, compactness, surface roughness, average intensity, and internal intensity standard deviation were obtained from the 3D OCT images. The complete spectral range (from 600 to 2100 cm^−1^) was used after fluorescence components were removed from the obtained Raman spectra, yielding an 85.9% classification accuracy for the five types of skin cells. While RS identified malignant cells with nearly 100% accuracy, the cellular-resolution OCT images clearly distinguished malignant cells from normal cells. Cell surface roughness, internal average intensity, and the standard deviation of internal intensity distribution were among the OCT image elements that were successful in separating keratinocytes from melanocytes as well as malignant cells from normal cells. It was possible to distinguish clearly between MM and keratinocyte-based cell carcinoma cells using the complete Raman spectra [109]. A trimodal system that combines OCT and optoacoustic modalities to provide accurate tumor depth delineation has been designed for quick, harmless in vivo skin cancer screening. It is further enhanced by a Raman spectroscopic modality that can identify the type of lesion, further extending the diagnostic capabilities of the system [112].

#### 4.3.4. Raman and Autofluorescence Spectroscopy 

When combined with autofluorescence, RS offers a way to evaluate biochemical alterations in tissues. This could be a useful addition to dermoscopy analysis, which is mostly used to evaluate tissue morphological features in skin neoplasms. Optical biopsy, which utilizes both Raman spectroscopy and autofluorescence, demonstrates a relatively high degree of accuracy in diagnosing skin cancer [56,115,116,117,118]. The in vivo feasibility of a portable, low-cost spectroscopy setup that integrates the analysis of Raman and autofluorescence spectra has been tested on a cohort of 56 patients (comprising 19 MM, 18 BCC, and 19 benign neoplasms), achieving an accuracy range of 89–100% in classifying skin neoplasms (differentiating MM from other neoplasms, BCC from other neoplasms, MM from BCC, and MM from benign neoplasms) [12]. A subsequent study by the same group, encompassing 617 skin neoplasm cases (615 patients, 70 MM, 122 BCC, 12 SCC, and 413 benign tumors), yielded ROC AUC values of 0.75 (95% CI: 0.71–0.79), 0.69 (95% CI: 0.63–0.76), and 0.81 (95% CI: 0.74–0.87) for the classification of (a) malignant versus benign tumors, (b) MM versus pigmented tumors, and (c) MM versus seborrheic keratosis, respectively. The ranges for the positive and negative predictive values were 20–52% and 73–99%, respectively [119]. Due to the high autofluorescence rate in the recorded spectra, the classification of MM and pigmented lesions showed the least effective performance. Autofluorescence is frequently interpreted as an incorrect signal in NIR Raman spectroscopy investigations, and strong autofluorescence rates mask low-intensity Raman peaks [12,120,121]. Melanin is the primary NIR fluorophore in human skin tissue. Given that MM and nevi frequently have a high melanin content, classifying MM and pigmented lesions using low-cost Raman devices becomes challenging due to the elevated autofluorescence background [120]. Through integrating spectral analysis with the examination of unique patient characteristics and causes of skin cancer progression, improved skin cancer diagnosis was accomplished. Combining spectral data within the 300–1800 cm^−1^ range with three patient factors (sex, age, and tumor localization) associated with skin cancer growth resulted in a statistically significant improvement in the classification of 413 benign and 204 malignant tumors, with the ROC AUC rising from 0.61 to 0.82, *p* = 2 × 10^−11^. A statistically significant improvement in the AUC from 0.61 to 0.79, *p* = 5 × 10^−7^, was also observed in the classification of 324 benign tumors and 157 malignant tumors using spectral data in conjunction with eight risk factors (sex, age, tumor localization, family history, personal history, sun exposure, size, and occupational hazards). Ultimately, through merging the spectral data with the three risk factors, the discrimination of 70 MM and 283 benign pigmented skin neoplasms was accomplished with a statistical boost from 0.71 to 0.83 (*p* = 0.02) [122].

#### 4.3.5. Raman and Laser-Induced Breakdown Spectroscopy

A hybrid multimodal instrument that records Raman and laser-induced breakdown spectroscopy (LIBS) is a powerful way to gain an extensive understanding of a wide range of samples that are common in modern living environments. Combining these methods can greatly increase the instrument’s adaptability and flexibility in handling the challenging requirements of real-world situations. In light of their complimentary ability to analyze tissue architecture, combined LIBS–Raman analysis and detection may improve the effectiveness of discriminating. The accuracy of the discriminating and classification of MM and normal formalin-fixed paraffin-embedded (FFPE) tissues was significantly enhanced through the integration of LIBS and Raman spectroscopy. A 100% discriminating accuracy between MM and normal samples was obtained using an extreme learning machine classification model; this result was repeated in the combined data, as well as the individual Raman and LIBS datasets [123].5. Future Directions

The future of Raman spectroscopy in skin cancer diagnostics is potentially riveting, with myriad advancements on the horizon. Technological advancements are expected to propel Raman spectroscopy into new dimensions of diagnostic efficiency and accuracy. Notably, enhancements in sensitivity, resolution, and real-time data acquisition are pivotal steps toward more effective skin cancer diagnostics [2]. 

Moreover, the integration of artificial intelligence with Raman spectroscopy is expected to augment the diagnostic accuracy and efficiency. Through AI algorithms, subtle spectral markers associated with neoplasms can be identified, enabling early diagnosis and personalized treatment plans owing to their unique ability to identify molecular signatures. This enables the characterization of specific patient conditions and the tracking of molecular changes over time. This information can guide personalized treatment planning and ensure that the most effective therapies are administered. Raman spectroscopy excels in early disease detection, enabling timely interventions, and distinguishes between benign and malignant lesions. Its minimally invasive nature allows for longitudinal monitoring, and real-time feedback during surgery aids precise tissue removal. Overall, Raman spectroscopy contributes to patient-specific data collection, facilitates tailored medical approaches, improves patient care, and monitors disease progression [2].

The coupling of Raman spectroscopy with other diagnostic modalities, such as optical coherence tomography, ultrasound, or magnetic resonance imaging (MRI), could provide the morphological and biochemical insights essential to a comprehensive assessment of skin lesions. Such multimodal diagnostic approaches could offer a more holistic view of skin tissues, aiding better-informed clinical decisions [124].

Furthermore, the field of nanotechnology, especially SERS, is believed to be a game changer in improving the sensitivity and specificity of Raman spectroscopy. The application of nanoparticles can significantly enhance Raman spectroscopic signals, thus enabling the detection of low-abundance molecular species indicative of early stage skin cancer [2]. 

Advancements in imaging techniques coupled with Raman spectroscopy are expected to evolve, providing high-resolution images along with molecular data. This synergy would allow for a deeper understanding and analysis of malignant and benign skin tissues, significantly aiding the early detection and diagnosis of skin cancer [124].

Raman spectroscopy opens up a promising future in advancing skin cancer diagnostics by enabling early detection, real-time analysis, and integration with other diagnostic technologies. Its potential to distinguish between different types of skin tumors further emphasizes its significance in the domain of skin cancer diagnostics. Raman spectroscopy can serve as an intraoperative guide during skin cancer excision. It is possible to lessen the amount of leftover tumor and avoid removing too much good tissue by clearly demarcating the tumor boundaries. Its ability to rapidly characterize tissue based on molecular composition enables surgeons to distinguish between cancerous and healthy tissue during the procedure. This real-time feedback guides surgeons in making precise decisions about where to cut or preserve tissue, reducing the risk of over-resection and ensuring that all cancerous cells are removed. Additionally, when no representative tumor tissue is initially found, Raman-guided biopsy can increase the accuracy of the biopsy and reduce the need for repeat stereotactic biopsy procedures [125]. 

Given its ability to differentiate early stage lesions, Raman spectroscopy can be employed in early screening tests for skin cancer [68]. Moreover, the Raman technique also holds value for radiation therapy [126]. There is also an emphasis on exploring patient-specific analyses by tailoring diagnostic approaches to individual patients, potentially providing more personalized diagnostic insights. The utilization of RS for the longitudinal monitoring of skin lesions over time to observe disease progression or regression and to monitor the response to treatment is considered a beneficial approach [70,127].

The development of portable and handheld Raman spectroscopy devices is a significant step toward point-of-care diagnostics, facilitating real-time analysis in clinical settings, which is indispensable for the early detection and diagnosis of skin cancer [59]. Tailoring the diagnostic approach to individual patients could potentially provide more personalized diagnostic insights, although further research and references are needed to elaborate on this aspect. The many advantages of multimodal methods are shown via the use of RS for the longitudinal monitoring of skin lesions over time to assess disease progression or regression and to monitor the response to treatment. Wearable or flexible SERS substrates for measuring human skin are a new development in SERS technology. The development of this sophisticated SERS sensor is a major step in improving the wearable sensing technology’s practicality and generalizability [5].

For the broad-scale implementation of Raman spectroscopy, multiple aspects need to be addressed, including cost, maintenance, personnel training, data analysis, and the duration of investigation. Raman spectroscopy mandates regular calibration and ongoing maintenance, which might escalate its cost [128]. Although obtaining data from samples through Raman spectroscopy is relatively simple, the analysis of such data requires advanced skills. This underscores the importance of training adept algorithm designers [129]. The quality of Raman spectroscopy in practical applications can be compromised by various factors, including the interference of fluorescence signals, sample heterogeneity, and sample degradation due to overexposure to laser light. Additionally, environmental factors, such as temperature fluctuations, humidity, and vibrations, can introduce noise into Raman spectra. To mitigate these challenges, researchers have employed techniques to control fluorescence, ensure sample homogeneity, and maintain stable environmental conditions. Preprocessing steps, such as baseline correction and noise reduction, are crucial for improving signal accuracy and interpretability. Furthermore, proper instrument calibration and regular maintenance are essential to prevent instrumental drift or misalignment and ensure the reliability of Raman measurements. Careful consideration of these factors, and appropriate measures to address them, are vital for obtaining accurate and meaningful data for practical Raman spectroscopy applications.

The future of Raman spectroscopy in skin cancer diagnostics is envisaged to include a blend of technological, methodological, and collaborative advancements that could revolutionize skin cancer diagnostics and patient care. Through global collaborations among researchers, clinicians, and policymakers, advancements and the global adoption of Raman spectroscopy in skin cancer diagnostics could be expedited, heralding a new era of enhanced diagnostic accuracy, early detection, and personalized patient care.

## 5. Conclusions

The future of Raman spectroscopy in skin cancer diagnostics is marked by significant advancements and potential innovations. Technological improvements are anticipated to enhance the diagnostic efficiency and accuracy of Raman spectroscopy, particularly through increased sensitivity, resolution, and real-time data acquisition. The integration of artificial intelligence with Raman spectroscopy is expected to augment diagnostic accuracy and efficiency, enabling early diagnosis and personalized treatment plans through the identification of subtle spectral markers and molecular signatures. This integration will facilitate the characterization of specific patient conditions and the monitoring of molecular changes over time. Furthermore, the combination of Raman spectroscopy with other diagnostic modalities like optical coherence tomography, ultrasound, or MRI is expected to provide comprehensive assessments of skin lesions by offering both morphological and biochemical insights. The field of nanotechnology, especially SERS, is seen as a critical factor in enhancing the specificity and sensitivity of Raman spectroscopy, enabling the detection of low-abundance molecular species indicative of early stage skin cancer. Advancements in imaging techniques coupled with Raman spectroscopy are also anticipated, which will aid in the early identification and diagnosis of cutaneous neoplasms by allowing a deeper understanding and analysis of both benign and malignant skin tissues. Additionally, there is a focus on developing patient-specific analyses and diagnostic approaches, as well as the use of Raman spectroscopy for the longitudinal monitoring of skin lesions to observe disease progression or responses to treatment. The development of portable and handheld Raman spectroscopy devices represents a significant step toward point-of-care diagnostics, enhancing the practicality of early detection and diagnosis of skin cancer. The emergence of wearable or flexible SERS substrates for measuring human skin marks an important development in SERS technology. However, the practical application of Raman spectroscopy faces challenges due to factors such as fluorescence signal interference, sample heterogeneity, and potential sample degradation due to overexposure to laser light, as well as environmental factors like temperature fluctuations.

## Figures and Tables

**Table 1:** Analytical techniques and their applications in skin cancer diagnosis and research.

Technique	Applications	Key Findings	Ref
Raman spectroscopy	Skin cancer detection	Higher precision in ex vivo detection compared to in vivo. Sensitivity and specificity values reported for BCC, MM, and SCC. Ex vivo imaging revealed spatial distribution of tissue structures, but was time-intensive.	[7,22,23,24,25,26,27,28,29,30,31,32]
Near-infrared Raman spectroscopy	Skin tissue biochemical analysis	Identified key biochemicals like actin, collagen, elastin, and triolein. Classification model differentiated normal tissues from BCC and MM tissues.	[32]
Raman spectroscopy	Skin cancer diagnosis	PCA and Euclidean distance distinguished Raman spectra of BCC and MM from normal tissues in vitro, achieving a high diagnostic accuracy.	[22]
Raman spectroscopy	BCC diagnosis	Identified spectral variances between normal skin tissues and BCC-affected tissues. Diagnostic algorithms based on PCA and Mahalanobis distance identified tissue types effectively.	[28,40,41,42,43,44,45,46]
Coherent anti-Stokes Raman scattering assessments	BCC imaging	Produced pseudo H&E stained ex vivo microscopic imagery of BCC human skin specimens.	[47]
Raman microspectroscopy	SCC molecular analysis	Identified decrease in collagen intensities and increase in DNA and lipid intensities in SCC lesions. Stimulated Raman scattering microscopy revealed pathological characteristics within SCC tissues.	[48]
Spatially offset Raman spectroscopy	NMSC diagnosis	Effective for diagnosing NMSCs prior to histopathological examination. Identified specific wavenumbers associated with SCC and BCC.	[49]
Raman spectroscopy	BCC and SCC lipid and collagen analysis	Identified spectral characteristics distinguishing BCC and SCC groups based on lipid and collagen content.	[49]
Raman spectroscopy	Skin condition differentiation	Enhanced diagnostic accuracy in distinguishing among skin conditions using normal-subtracted preprocessing on Raman spectra.	[50]
Raman spectroscopy	Skin lesion diagnosis	Utilizing LASSO-based wavenumber selection with PCA and GDA analysis enhanced diagnostic performance in a merged cohort of skin lesions.	[51]
Raman spectroscopy	Differentiation of BCC, MM, and other conditions	MCR-ALS technique applied to analyze in vivo Raman spectra for differentiating BCC, MM, and other conditions. ROC AUC values reported for various discrimination models.	[52]
Raman spectroscopy imaging with MCR-ALS algorithm	MM and dysplastic nevi analysis	MM, dysplastic nevi, and compound nevus tumors. High sensitivity and specificity in identifying dysplastic nevi lesions and differentiating dysplastic nevi and MM samples.	[53]
Raman spectroscopy with silver-coated silicon nanowires	Genomic DNA differentiation in MM	Facilitated differentiation between healthy and cancerous genomic DNA extracted from normal human skin cells and MM cells. Achieved discrimination with no false-negative detections and a minimal false-positive rate of less than 2%.	[54]
Advanced Raman techniques (SRS and CARS)	Ex vivo characterization of NMSCs	Employing a multimodal methodology that includes coherent anti-Stokes Raman scattering, second harmonic generation, and two-photon excited fluorescence, distinctive morphological attributes of BCC and SCC were identified through H&E staining. Differences in coherent anti-Stokes Raman scattering imagery between BCC and SCC were noted, providing insights into lipid metabolism, bioenergetics, and tumor–stroma interactions. The amalgamated information aids in distinguishing healthy skin from NMSCs and between BCC and SCC.	

Abbreviations: AUC, area under the curve; BCC, basal cell carcinoma; CARS, coherent anti-Stokes Raman spectroscopy; GDA, general discriminant analysis; H&E, hematoxylin and eosin; LASSO, least absolute shrinkage and selection operator; MCR-ALS, multivariate curve resolution-alternating least square; MM, malignant melanoma; NMSC: non-melanoma skin cancer; PCA, principal component analysis; ROC, receiver-operating curve; SCC, squamous cell carcinoma; SRS, stimulated Raman scattering.

## Data Availability

Not applicable.
